# Incidental Phaeochromocytoma on Staging PET-CT in a Patient with a Sigmoid Tumour and Situs Inversalis Totalis

**DOI:** 10.1155/2014/645462

**Published:** 2014-07-08

**Authors:** M. R. Boland, A. J. Lowery, S. Walsh, D. Beddy, R. S. Prichard, D. O'Shea, S. J. Skehan, E. W. McDermott

**Affiliations:** ^1^The Department of Endocrine, St Vincent's University Hospital, Elm Park, Dublin 4, Ireland; ^2^The Department of Colorectal Surgery, St Vincent's University Hospital, Elm Park, Dublin 4, Ireland; ^3^The Department of Endocrinology, St Vincent's University Hospital, Elm Park, Dublin 4, Ireland; ^4^The Department of Radiology, St Vincent's University Hospital, Elm Park, Dublin 4, Ireland

## Abstract

An adrenal “incidentaloma” is defined as an unexpected finding on radiological imaging performed for unrelated indications. Improvements in radiological technology have seen a dramatic increase in this phenomenon. We report the unique case of a 60-year-old female presenting with a 6-month history of abdominal pain, altered bowel habit, and rectal bleeding. Her past medical history included situs inversus totalis and a patent ductus arteriosus. Colonoscopy revealed an ulcerated tumour in her sigmoid colon. Staging PET-CT confirmed a sigmoid tumour and also identified a large heterogenous enhancing FDG-avid right adrenal mass. Biochemical testing/MIBG imaging confirmed a right adrenal phaeochromocytoma. Hypertension was controlled and excision was performed via a transperitoneal laparoscopic adrenalectomy, in the left lateral decubitus position. Uniquely, liver retraction was not required due to its position in the left hypochondrium. Histology confirmed a benign 46 mm phaeochromocytoma. Subsequent uncomplicated sigmoid colectomy/right salpingo-oophorectomy for a locally advanced colonic tumour was performed with adjuvant chemotherapy. This case highlights the importance of accurately identifying functioning adrenal tumours before elective surgery as undiagnosed phaeochromocytomas carry significant intraoperative morbidity/mortality. Right adrenalectomy was made easier in this patient by the liver's unique position. Uncomplicated colorectal resection was made possible by combined preoperative functional/anatomical imaging.

## 1. Introduction

The increasing incidence of adrenal incidentalomas identified by improved radiological techniques has been mirrored by an increase in the incidence of asymptomatic phaeochromocytomas [1]. The treatment of these functional lesions can be challenging especially in the rare setting of situs inversus totalis.

## 2. Presentation of Case

We report the case of a 60-year-old female who presented with a six-month history of intermittent crampy abdominal pain associated with an alteration in her bowel habit and two episodes of rectal bleeding. Her background medical history included situs inversus totalis combined with a congenital patent ductus arteriosus, mitral regurgitation which required placement of a metallic mitral valve, complete heart block requiring placement of a dual chamber pacemaker, and dilated cardiomyopathy with an ejection fraction of 24%. She also had a longstanding history of hypertension. Her medications included telmisartan 20 mg daily per oral (PO), bisoprolol 2.5 mg daily PO, furosemide/amiloride 40 mg/5 mg daily PO, and warfarin 6 mg daily PO or as per regular international normalised ratios. She was an ex-smoker and reported no history of familial syndromes.

**Figure 1:**
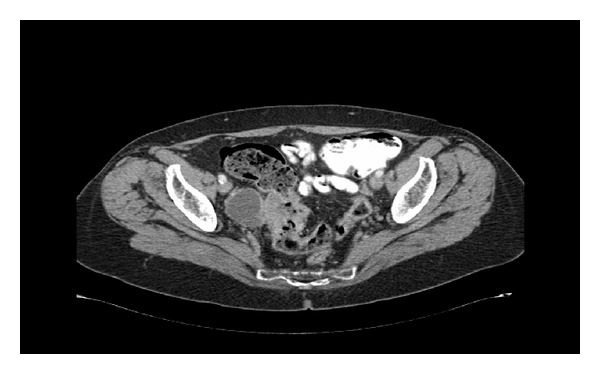
Sigmoid tumour with an exophytic portion that abuts the right fallopian tube.

**Figure 2:**
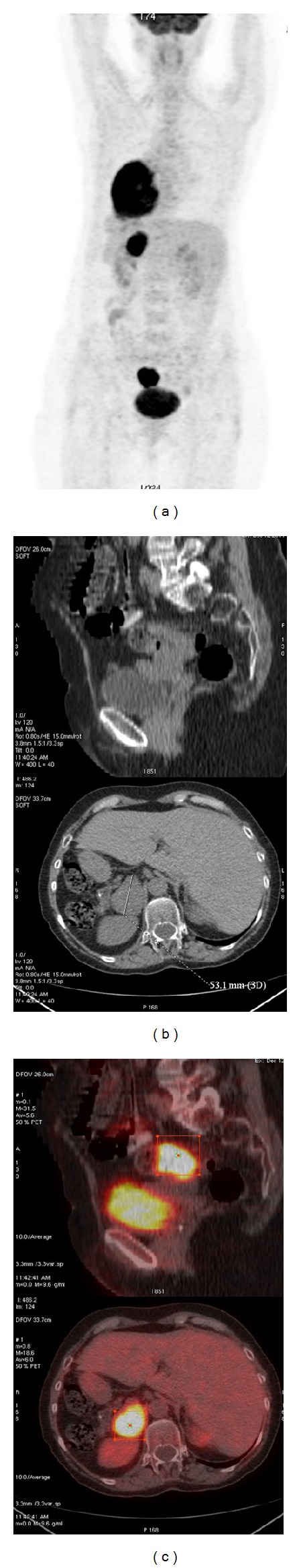
PET-CT sections showing the FDG-avid right adrenal mass.

The patient underwent a colonoscopy which showed an ulcerated sigmoid lesion at 50 cm. Biopsy was not performed due to the patient's ongoing anticoagulation. She proceeded to have a staging Positron Emission Tomography-Computed Tomography (PET-CT) scan which confirmed the presence of a FDG-avid sigmoid lesion with an exophytic component that abutted the right fallopian tube and likely represented a carcinoma with adjacent invasion (Figure 1). The PET-CT scan also identified a 5.3 × 3.8 cm heterogenous enhancing FDG-avid right adrenal mass (Figures 2(a), 2(b), and 2(c)).

The patient had no history of flushing, palpitations, or diaphoresis and her haematological investigations were normal. Blood and urine investigations were performed to assess whether this lesion was functionally active. A biochemical diagnosis of a phaeochromocytoma was made with a 24-hour urine collection for catecholamine which revealed an elevated urinary noradrenaline of 5630 nmol/d (n.v. 90–500 nmol/d) and elevated urinary vanillylmandelic acid of 66.3 mcmol/d (n.v. < 33 mcmol/d).

**Figure 3:**
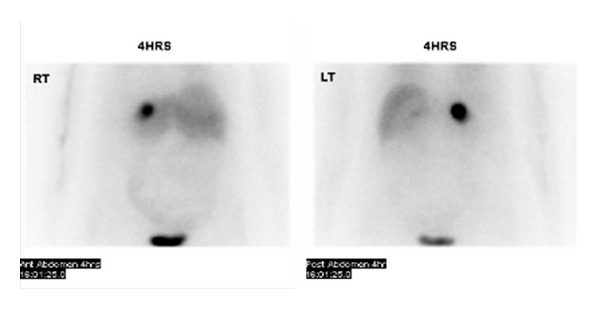
MIBG scan showing increased uptake in the right adrenal gland.

An MIBG scan was subsequently performed with I^123^ and demonstrated avid tracer uptake in the right adrenal mass with no evidence of any abnormal uptake elsewhere, confirming the adrenal lesion as a phaeochromocytoma (Figure 3).

The patient was scheduled for a laparoscopic right adrenalectomy prior to a sigmoid colectomy, appendicectomy, and right salpingo-oophorectomy. Oral anticoagulation with warfarin was discontinued and bridging therapeutic low molecular weight heparin was commenced. Hypertension was controlled preoperatively by intravenous phenoxybenzamine (alpha blockade) commenced two days prior to the operation and given as a daily infusion at a dose of 0.5 mg/kg in 400 mLs of normal saline over 2–4 hours. The angiotensin receptor blocker, telmisartan, was ceased preoperatively. Volume expansion in the form of normal saline was commenced two days preoperatively at a rate of 1000 mL per 12 hours and the patient's blood pressure was monitored at four hourly intervals.

**Figure 4:**
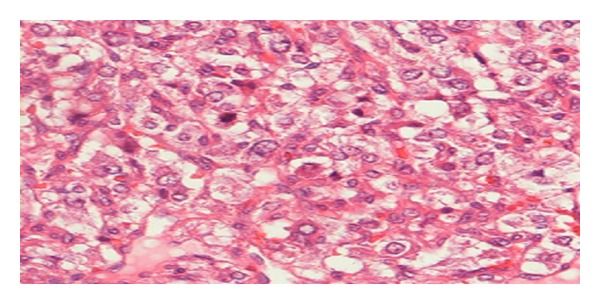
Features above include granular eosinophilic cytoplasm and moderate nuclear pleomorphism, consistent with phaeochromocytoma.

The patient underwent a laparoscopic right adrenalectomy in the left lateral decubitus position via a transperitoneal approach. Amoxicillin, gentamicin, and metronidazole were given in the perioperative period. There were no intraoperative complications. She was electively admitted to the intensive care unit in the postoperative period where she transiently required inotropic support although her blood pressure was stable throughout. The patient made an uneventful recovery and was discharged on day 3. Histological examination of the resected specimen revealed a 46 mm encapsulated cellular lesion. Cellular characteristics identified included granular eosinophilic cytoplasms and moderate nuclear pleomorphism, consistent with phaeochromocytoma, as shown in Figure 4. Immunohistochemistry for mismatch repair proteins showed no loss of MLH1, PMS2, MSH2, and MSH6.

She was readmitted to hospital 14 days later and underwent a high anterior resection with an en bloc right salpingo-oophorectomy to remove the locally advanced colonic tumour. Histological examination of the resected specimen revealed an invasive moderately differentiated T4 N0 M0, Dukes Stage B adenocarcinoma invading the adherent fallopian tube mucosal lining. The patient made an excellent postoperative recovery. She did not receive adjuvant chemotherapy due to her comorbidities and remains disease free at 20 months follow-up.

## 3. Discussion

Situs inversus totalis (SIT) represents a complete left to right side transposition of the asymmetrical thoracic and abdominal organs and incorporates dextrocardia. It is estimated to occur in between 1 : 5,000–20,000 adults [2]. It is frequently complicated by concurrent abnormalities in the cardiovascular system, as was evident in this case, and the hepatobiliary system. Congenital heart disease is estimated, in one study, to complicate up to 70% of cases of SIT with dextrocardia [3] and was evident in our patient in the form of a patent ductus arteriosus. Interestingly, the presence of phaeochromocytomas in the setting of SIT is extremely rare [4, 5]. Whilst phaeochromocytomas occur commonly in the presence of genetic conditions such as multiple endocrine neoplasia type 2 and von Hippel-Lindau diseases, there is little evidence to suggest that it is genetically related to SIT and likely occurs as a sporadic unrelated entity.

This case presented a unique management challenge. The management of phaeochromocytomas in any patient can be difficult but in the setting of SIT meticulous and calculated preoperative planning is imperative. Alpha blockade, with oral phenoxybenzamine, facilitates volume expansion, controls blood pressure, and reduces intraoperative hypertensive episodes [6]. In the presence of complex cardiac morbidities BP and fluid status should be monitored closely in the perioperative period [7]. Side effects to watch for include headache, palpitations, orthostatic hypotension, and tachycardia [8]. Patients are deemed ready for surgery when supine arterial blood pressure is not greater than 160/90, orthostatic hypotension is not more than 80/45, and ECG monitoring shows no ST segment or T wave changes for 2 weeks. If necessary, beta blockade can also be commenced to prevent persistent tachycardia and arrhythmias but is contraindicated until adequate alpha blockade has been achieved, to avoid unopposed alpha vasoconstriction and severe hypertension. Postoperatively, glycemic control should be monitored closely, as the sudden withdrawal of catecholamines can increase insulin sensitivity and cause severe hypoglycaemia [9].

It is impossible to ascertain how long the phaeochromocytoma had been present for but certainly it is possible that longstanding excess catecholamine secretion may have contributed to the patient's cardiac morbidity and more specifically to the development of significant dilated cardiomyopathy, which has been well described in the literature [10].

As functional imaging modalities continue to improve, incidental adrenal masses (incidentalomas) are becoming more frequent [1], especially as the classic signs and symptoms of lesions such as phaeochromocytomas can often be masked by underlying cardiac problems as well as medication and this was likely evident in this case. When these lesions are identified, clinical assessment and hormonal/functional investigations need to be performed in a timely manner [11]. These include a 1 mg overnight dexamethasone suppression test to assess for evidence of abnormally increased glucocorticoid production, urinary metanephrine ± catecholamine levels which are usually elevated in the presence of a phaeochromocytoma, and erect plasma aldosterone : renin ratio to assess for hyperaldosteronism. Distinguishing between phaeochromocytomas and paragangliomas can be difficult as extra-adrenal paragangliomas almost invariably secrete noradrenaline whereas phaeochromocytomas secrete both noradrenaline and adrenaline with a propensity to the latter. Nuclear imaging, such as MIBG scans, can also be used to elucidate the nature of an incidentaloma and exclude multiple extra-adrenal lesions. Indications for surgery on incidental adrenal masses include all lesions > 6 cm, lesions 4–6 cm with certain radiological features, any mass with features suspicious for adrenal malignancy or hyperfunctionality, or any lesion that changes on follow-up to become hyperfunctional, malignant, or increasing in size by >1 cm [11]. A laparoscopic approach is now recommended as it has been shown to reduce morbidity and length of hospital stay [12]. Notably, up to one-third of patients have concomitant, or a history of, malignancy with phaeochromocytomas representing about 10% of this group [13].

The surgical approach was also critically important, especially in the setting of SIT. Laparoscopic adrenalectomy has become the gold standard of care for small nonmalignant appearing phaeochromocytomas [14]. Whilst a laparoscopic approach in this case was much more challenging on account of organ position, access to the right adrenal gland was actually easier in this setting as the need for liver retraction was absent due to its presence on the left side of the abdomen. Performing the operation laparoscopically was also beneficial as it allowed the second procedure to resect the patient's sigmoid tumour to be performed after an interval of 2 weeks. This would not have been possible if the adrenalectomy had been performed by an open technique. Notably, up to 70% of patients with SIT and dextrocardia have some form of congenital heart disease and so formal cardiac investigations may be useful preoperatively [15]. Furthermore, Kartagener's Syndrome and primary ciliary dyskinesia are also more prevalent in SIT patients which again needs to be accounted for when planning preoperatively. Finally, the surgeon needs to be extremely careful to avoid errors that occur with SIT due to reverse side anatomy.

## 4. Conclusion

In conclusion, this case demonstrates the unique surgical challenge one faces when dealing with a patient with serious pathologies in the setting of SIT. Careful planning, multidisciplinary input, and a unique approach to surgery, given the anatomical anomalies present, are paramount to a successful outcome.
